# Improving Meropenem Quantification in a Compact SERS-Based Centrifugal Microfluidic Platform: Toward TDM of Antibiotics in ICU

**DOI:** 10.1021/acs.analchem.4c06902

**Published:** 2025-03-25

**Authors:** Martyna A. Pytlarz, Gohar Soufi, Isidro Badillo-Ramírez, Laura Serioli, Roman Slipets, Anders Perner, Anja Boisen

**Affiliations:** † Center for Intelligent Drug Delivery and Sensing Using Microcontainers and Nanomechanics (IDUN), Department of Health Technology, Technical University of Denmark, Lyngby, Kongens 2800, Denmark; ‡ Department of Intensive Care, Copenhagen University Hospital−Rigshospitalet, Copenhagen 2100, Denmark; § Department of Clinical Medicine, University of Copenhagen, Copenhagen 2200, Denmark

## Abstract

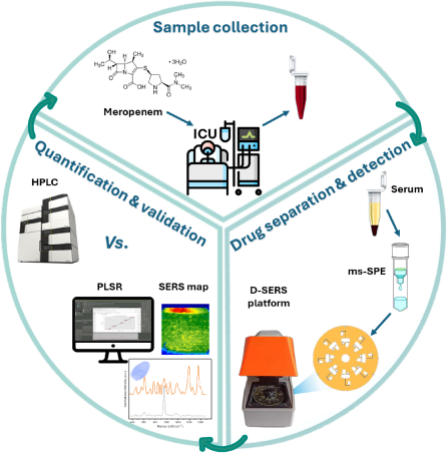

Infections are the leading causes of death, especially in intensive care units (ICUs), necessitating immediate and optimal antibiotic treatment with proper monitoring of the drug dosage. Present analytical techniques measuring antibiotic levels result in a long lag time for dose adjustments. Therefore, introducing versatile techniques that quickly quantify antibiotic levels in a patient’s blood is essential. We developed a novel approach to advance the automation of label-free surface-enhanced Raman spectroscopy (SERS) in a centrifugal microfluidic setting (D-SERS device) to improve the quantification of meropenem (MER) in serum samples. The D-SERS device consists of a microfluidic disc cartridge, a spin motor, and an integrated Raman spectrometer module. In our assay method, we implemented a serum cleanup step, employing a monospin solid-phase extraction (ms-SPE) column, which was coupled with the D-SERS device. The MER label-free detection was performed on-disc by SERS scanning of a Ag nanopillar substrate integrated into the disc cartridge. We identified that coupling ms-SPE to the D-SERS device led to significant improvement in a signal-to-noise ratio and sensitivity. Chemometrics algorithms, such as partial least squares regression (PLSR), were implemented on a large data set for SERS analysis, allowing LoD and LoQ values of 12.12 and 36.37 μM, respectively. We compared the performance of our D-SERS device to that of a commercial Raman system, demonstrating its efficiency and reliability. Moreover, the D-SERS device was validated against HPLC employing samples from ICU patients, showing a good correlation (*R*^2^ = 0.8, *p* < 0.05) with a bias of 14.3 μM overestimation. The whole D-SERS system is compact and easy to operate, and results are obtained within 15–20 min, supporting its clinical feasibility for point-of-care therapeutic drug monitoring of antibiotics.

## Introduction

Sepsis is characterized by a dysregulated immune response to infections, life-threatening multiple organ failure, and high mortality. It is a global health challenge with approximately 11 million deaths annually worldwide.^1^ Immediate administration of appropriate antibiotics is vital for patient survival, especially in intensive care units (ICUs), but optimal antibiotic dosing remains a challenge.^2^ Current practices show that only about 50% of patients receive a correct dose, with many patients being overdosed (affecting approximately 26% of patients) and underdosed (affecting around 31%).^3,4^ Overdosing contributes to serious adverse effects such as renal failure and neurotoxicity, while underdosing fosters antibiotic resistance, leading to prolonged hospital stays, increased costs, and increased mortality.^5^

Meropenem (MER) is a broad-spectrum beta-lactam antibiotic that applies to a wide range of Gram-positive and Gram-negative bacteria and is regularly used for ICU patients with sepsis. Beta-lactams exhibit time-dependent antimicrobial activity. MER is a drug that requires strict monitoring in sepsis patients, ensuring optimal therapeutic outcomes while mitigating the risk of adverse effects.^6,7^ MER’s pharmacokinetic properties, including its predominantly renal clearance, variability in interpatient clearance rates, and the potential for altered pharmacokinetics in critically ill populations, indicate that individualized dosing strategies may reduce the risk of under- and overdosing.^7,8^

Therapeutic drug monitoring (TDM) of antibiotics has the potential to improve dosing and, thereby, outcomes for patients with sepsis. TDM measures and analyzes drug concentrations in a patient’s body fluids, mainly in blood, serum, or plasma.^9−11^ Advancing the TDM implementation of antibiotics can allow the identification of the most effective treatment regimens and minimize the risk of failure, toxicity, or adverse drug reactions.^12^ TDM of the MER may also reduce the risk of bacterial resistance. The commonly administered dose is 1 g every 6 or 8 h in critically ill patients, and patients’ blood concentrations vary from 20 to 450 μM.^7^

MER quantification for TDM in current clinical practice typically involves utilizing liquid chromatography techniques, mainly high-performance liquid chromatography (HPLC).^13^ While HPLC offers notable benefits in sensitivity, specificity, reproducibility, and simultaneous analyte analysis, it presents certain drawbacks. These include the need for expensive and laborious hands-on equipment, the requirement of highly skilled staff, substantial solvent usage (resulting in increased waste), and, therefore, a longer sample-to-answer time duration. TDM of MER is, consequently, rarely used in current practice. Hence, implementing novel devices offering high sensitivity, simplicity, and rapid drug concentration values could significantly enhance TDM for MER and other antibiotics. Furthermore, the lack of immunoassay kits for detecting MER (or other beta-lactam antibiotics) underscores the urgency of introducing alternative drug detection methodologies.

Surface-enhanced Raman spectroscopy (SERS) is a powerful technique with potential analytical performance for TDM applications of several relevant drugs in clinical use.^10,11,14−16^ SERS provides both sensitive and specific analyte fingerprint recognition due to the plasmonic enhancement of vibrational modes of molecules absorbed on or near the surface of metallic nanostructured materials.^17^ Commonly, SERS substrates involve metallic nanostructured types, normally based on Ag or Au, which can be in a colloidal or solid arrangement, in which each solid type provides different advantages but also some limitations. Solid SERS substrates, like ordered nanopillars, have shown benefits due to their reproducibility and large-scale production, showing a wide range of applications for highly sensitive detection and quantification of diverse types of molecules, including therapeutic drugs like oncological, antiepileptic, and antibiotics.^10,16,18,19^

Label-free SERS is a straightforward approach for molecular identification through the unique analyte molecular Raman fingerprint.^18^ However, to perform high sensitivity and specific analyte quantification under label-free SERS, an effective interaction between the metallic surface and the target analyte is needed.^20^ In this sense, the implementation of optimal matrix cleanup procedures or devices that can be coupled to the SERS assay can improve label-free drug quantification in complex matrices. Existing approaches with label-free SERS involving the use of biological samples such as blood, serum, or saliva imply the optimal implementation of one or several cleanup methods. Biological sample complexity can be lowered by introducing protein precipitation (PP), ultrafiltration (UF), liquid–liquid extraction, or solid-phase extraction (SPE) methods.^14,15,21,22^

One of the strategies to improve the label-free SERS quantification of analytes in biological samples is using the SERS nanopillar substrate as a filter, called nanopillar-assisted separation (NPAS).^14,22,23^ This separation can be achieved manually or with integrated devices containing a sample cleanup module coupled to a SERS setting unit. Compact and integrated platforms in a microfluidic design might allow better control of depositing the sample on the SERS substrate, making for more accurate quantification.^24−26^

Centrifugal microfluidic devices containing a SERS unit, usually called lab-on-a-disc SERS (L-o-D-SERS) or centrifugal disc SERS (D-SERS), are promising settings for automated label-free SERS analyte detection.^22,23,27^ In conventional microfluidic devices, the fluid sample is propelled through microchannels with pumps, while in centrifugal microfluidics, the fluid movement is achieved by inertial forces that act on fluids caused by the rotation of the disc platform.^28^ Additional advantages of automated centrifugal microfluidic devices include the use of very low volumes of samples (fewer than 30 μL), no need for external pumps, controlled handling of samples and solutions, short time analysis, and the ability to analyze several samples in the same unit at the same time.^28^

In this work, we aim to advance the development and implementation of an integrated label-free SERS setup, consisting of centrifugal microfluidics, for MER quantification in human serum toward a simple and efficient approach for TDM in ICU.

**Figure 1:**
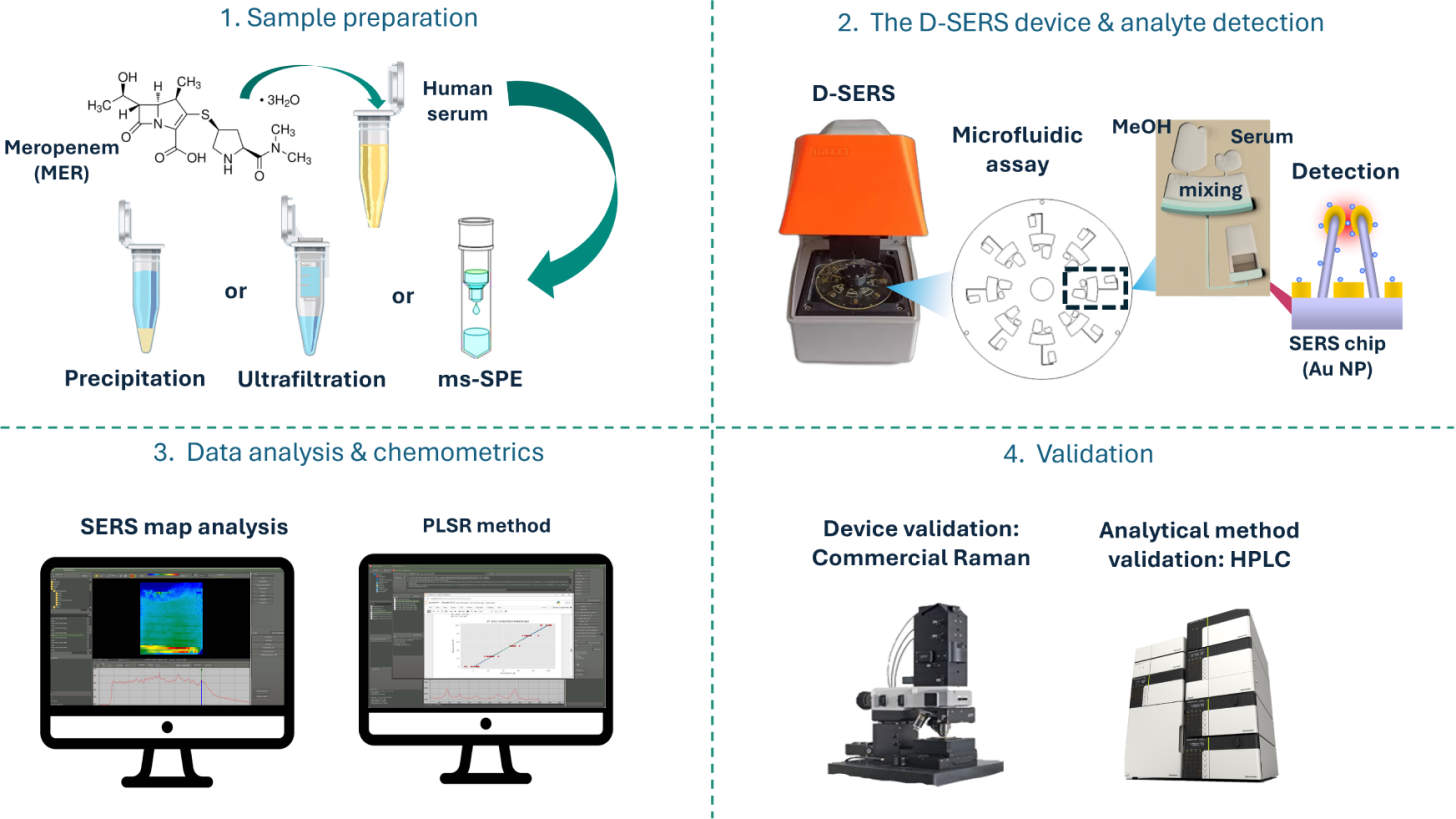
General procedure of MER quantification through label-free SERS: 1) sample collection and preparation, including PP on disc, UF, and ms-SPE off disc. 2) Utilizing the D-SERS platform for centrifugation and analyte migration toward the SERS nanopillar chip, employing fully automated software, and consecutive mapping of the SERS chip. 3) Advanced data analysis, based on chemometrics algorithms, of the collected spectral data set for MER quantification. 4) Validating the D-SERS device with a commercial Raman system and against HPLC for accuracy and assay analytical validation.

In this development, we implement several strategies in serum cleanup with PP, UF, and mono spin column SPE (ms-SPE) to effectively separate MER from human serum (step 1 in Figure 1). The separated analytes were further evaluated on the in-house developed centrifugal microfluidic device, containing a solid Ag NP substrate (the D-SERS device), to identify the most optimal cleanup method, based on the performance of sensitivity and specificity detection of MER (step 2 in Figure 1). MER quantification was further implemented through robust SERS spectral data analysis methods, based on chemometric algorithms, to improve its sensitivity and accuracy in commercial human serum and clinical samples (step 3 in Figure 1). Subsequently, we first validated our D-SERS device against a commercial Raman system, and then, our SERS assay was validated against HPLC to ensure the reliability and accuracy of MER quantification in clinical patient samples. This research endeavors to streamline antibiotic analysis processes with automated label-free SERS, providing a fast and reliable analytical tool for point-of-care applications in ICU settings.

## Experimental Section

### Chemicals and Materials

MER (certified reference material), commercial human serum (human male AB plasma), methanol (≥99.9%), and PBS (pH 7.4) were purchased from Sigma-Aldrich (St. Louis, MO, USA). Centrifugal filter units (0.5 mL) with 3 and 10 kDa pore sizes were purchased from Amicon Ultra, Merck Millipore Ltd. IRL. Commercially available monospin solid-phase extraction (ms-SPE) C18 columns (MonoSpin L) were purchased from GL Sciences. Detailed solution preparation methods can be found in Method M-S1.

### SERS Nanopillar Substrate Fabrication

Silver and gold nanopillars (NPs), Ag and Au NP SERS substrates, were fabricated following a previously reported method,^14^ consisting of etching and metal deposition steps. Method M-S2 presents a detailed description of the substrate fabrication. After production and before use, SERS chips were stored under vacuum to prevent contamination.

### Design and Fabrication of Microfluidic Discs

Microfluidic disc design and optimization were performed by using computer modeling. The disc consisted of eight units, each containing four chambers divided into two loading chambers, a mixing chamber, and a sensing chamber, where the SERS chip substrate is placed (see Figure S1 for schematic design and unit distribution). Detailed information on disc fabrication is provided in Method M-S3.

### Compact Raman Spectrometer with an Incorporated Spin Motor

The Raman device was homemade, consisting of a compact spectrometer module (Wasatch Photonics, USA), a single-mode laser at 785 nm wavelength, and standard sampling optics for an approximately 70 μm laser spot size. A full description of the compact Raman device and its schematic representation is shown in Method M4 and Figure S2, respectively. The system combines a camera for sample scanning, a motorized spinning mechanism to control the microfluidic disc, and a Raman mapping module for acquiring spectral data. The entire system is controlled by custom software developed using Delphi RAD Studio (Embarcadero Technologies, USA) and Python, which manages the coordination between the camera, spinning motor, and Raman spectrometer.

### Raman and SERS Analysis with a Commercial Spectrometer

Raman and SERS analysis was performed with a commercial instrument, the WITec Alpha 300R microspectrometer (Oxford Instruments). All details about measurements are described in Method M-S5.

### Sample Pretreatment Methods

For the label-free SERS analysis of the MER, three common sample pretreatment methods were employed: PP, UF, and ms-SPE (a schematic of the procedure is shown in Figure S3), which allowed the cleanup of human serum before SERS measurement. Conditions and optimization of each method are presented in Method M-S6.

### On-Disc Sample Separation and SERS Analysis

Analytes collected from the sample pretreatment methods were transferred to the centrifugal microfluidic disc for extra analyte separation on the SERS substrate due to the nanopillar filtering effect described in previous works.^14,23^ Serum and ultrafiltered serum were introduced with a micropipet into the (serum) loading chambers. MeOH was injected into the solvent chamber, and both solutions were mixed in the mixing chamber (Figure 1, step 2 or Figure S1), and then moved, by centrifugation, to the SERS sensing chamber. In the case of solutions from ms-SPE, they were injected directly into the mixing chamber (Figure S1), without adding MeOH, and then centrifuged to the SERS substrate. Centrifugal microfluidic conditions and the spinning program for centrifugal automation are described in detail in Method M7 and Figure S4.

### Patient Samples and HPLC Measurements

Twenty-seven whole blood samples from anonymous ICU patients under routine MER dosage were collected as leftovers from routine blood gas analysis (safePICO Aspirator, Radiometer, Brønshøj, Denmark) at Rigshospitalet, Copenhagen, Denmark. Initially, serum was separated from red blood cells via centrifugation for 5 min at 5000 rpm. Due to a reduced sample volume, 300 or 500 μL of the separated serum was diluted with commercial serum to get a final volume of 1500 μL for analytical correlation. Then, MER was separated by employing the ms-SPE column, as described above, and eluted analytes were used for HPLC (see HPLC measurement conditions in Method M-S8) and D-SERS analysis.

### Data Analysis

To ensure robust and reliable measurements, at least three SERS substrates (five for calibration curves) were employed in the D-SERS device and measured under identical experimental conditions. Data acquisition and real-time processing, including Raman spectral mapping, were managed within a custom software environment. The analytical process involved two main steps. Initially, spectra were preprocessed using a custom software tool, SERSanalyser (Figure S5). This preprocessing involved cropping each spectrum, performing background correction, and averaging the top 20% peak intensities at ∼1559 cm^–1^ across the pixels on each map (SERSanalyser). Subsequently, partial least squares regression (PLSR) was performed by using both custom-made software and the PLS toolbox in MATLAB (2021b) to enable accurate quantification of target analytes. All figures of merit for the PLSR model were calculated using the MVC1 toolbox. The integrated approach, which leverages Python-based tools and Jupyter Notebook (Figure S6) for statistical analysis, allows for precise and efficient chemical analysis in a miniaturized format, suitable for rapid and on-site testing. Detailed descriptions of the data analysis procedures are provided in Method M-S9.

## Result and Discussion

### Raman and SERS Characterization

**Figure 2:**
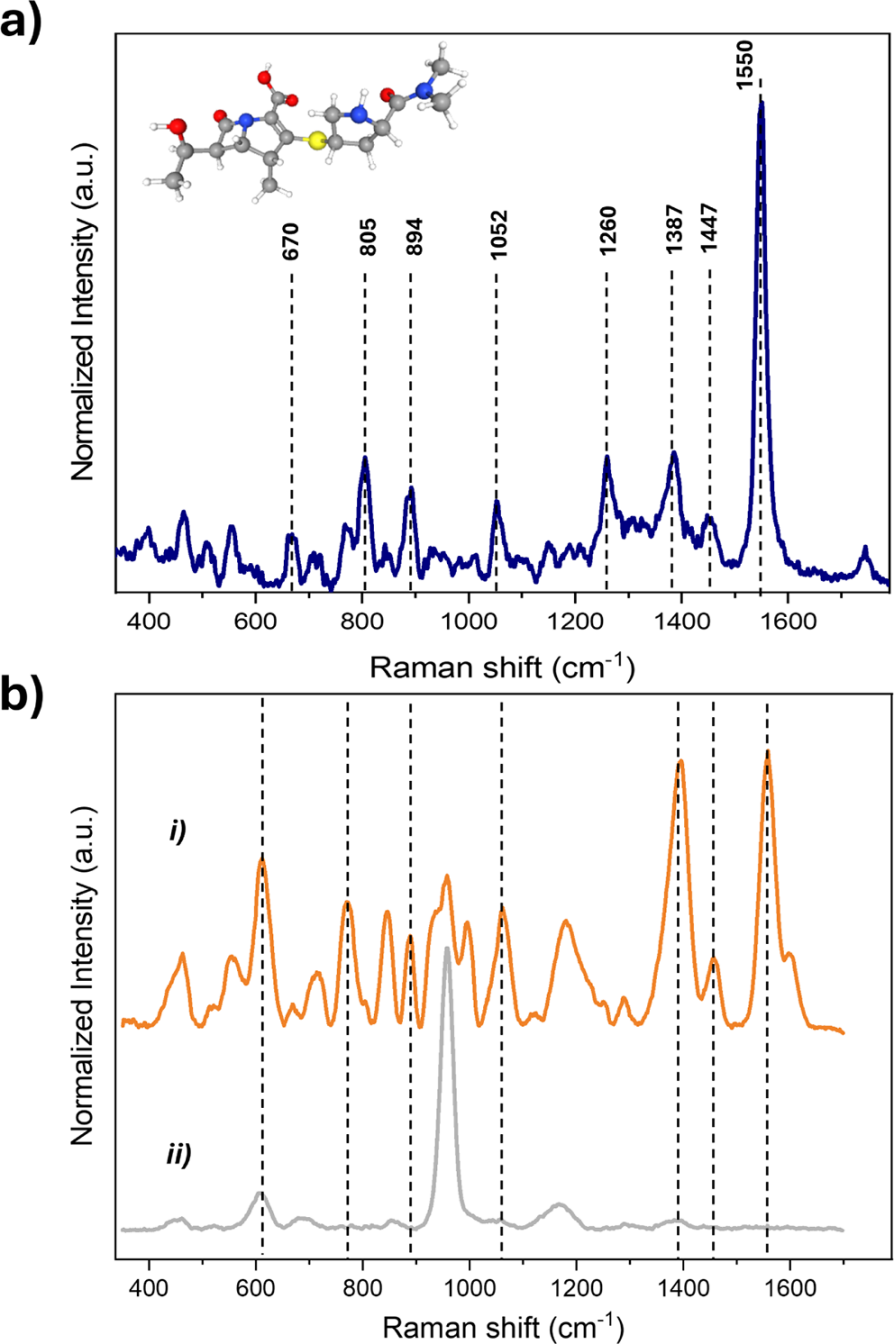
Raman and SERS Characterization of MER. (a) Raman analysis, indicating the main vibrational modes characteristic of the molecule (inset). (b) SERS spectrum acquired on a Ag-based NP substrate (orange spectra), compared with the Ag background substrate (gray); dotted lines indicate the corresponding MER bands correlated to the Raman spectrum.

The Raman and SERS spectra of MER were acquired and analyzed first before proceeding to its quantification. Figure 2a shows the MER reference Raman spectrum acquired in its crystalline form. The band identification shows a good correlation with the expected vibrational modes of the MER chemical structure (see inset in Figure 2a) and with previously published data.^29^

Distinctive bands of β-lactam-type antibiotics are found at positions 709, 769, and 805 cm^–1^, which are mainly attributed to the ring breathing mode of the β-lactam ring, while the band at 670 cm^–1^ is assigned to the pyrrolidone ring deformations. Bands at 894 and 1052 cm^–1^ are related to the C–C stretching in the β-lactam ring, while the band at 1387 cm^–1^ is attributed to the C–N stretching in the pyrrolidine ring. The most intense band at 1550 cm^–1^ is assigned to C=C stretching in the β-lactam ring. The considerably high intensity of this band can be attributed to the polarizability of the sulfur group adjacent to it.^30^ A detailed Raman band assignment is presented in Table S1.

The SERS characterization of MER, which has not been shown before in the literature, was performed by employing both Ag and Au NP-type substrates, and the corresponding spectra are shown in Figures 2b and S7, respectively.

The set of bands obtained with the Ag substrate (Figure 2b, orange line) shows a good correlation with the majority of bands identified from the Raman spectrum (Figure 2a). In this case, the most intense bands at 1387 and 1556 cm^–1^, assigned to combined modes of C—C, C–C, and C–N in the β-lactam ring, are clearly distinguished from the Ag substrate background (Figure 2b, gray line). Slight band shifting in some wavenumber positions, e.g., 1550 to 1556 cm^–1^, might be reflected from the preferred molecular orientation and adsorption on the metallic surface. Identification of this mode as the most intense band suggests that MER might be perpendicular to the Ag surface and adsorbed through the β-lactam ring, in which the carboxylic group might stabilize this interaction.^30^ Additional bands of medium to low intensity, at 1052, 894, and 771 cm^–1^, are also identified at wavenumber positions similar to those assigned in the Raman spectrum (dotted lines in Figure 2b). A detailed SERS band assignment is given in Table S1.

On the other hand, the SERS characterization with the Au substrate (Figure S7) shows a set of bands that are barely distinguishable from the Au SERS spectra background, suggesting that MER is very low SERS active on Au-based surfaces. Therefore, for the development of the analytical assay in this work, we employed the Ag NP as the main SERS substrate.

### Performance of Sample Preparation Methods

**Figure 3:**
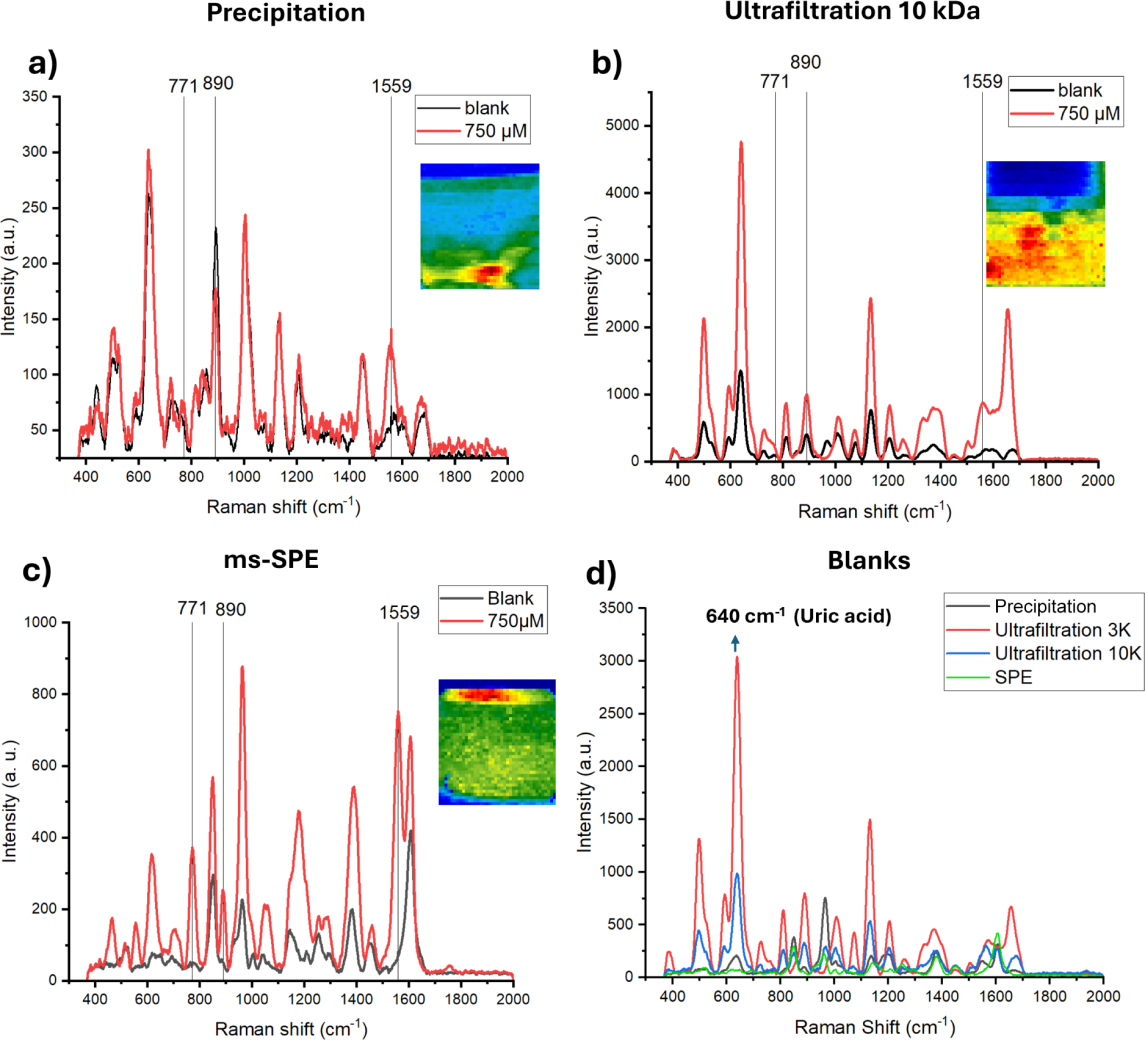
Comparison of the MER spectral profile and localization in the SER chip after performing the different sample pretreatment methods: (a) PP, (b) UF, and (c) ms-SPE. (d) Comparison of the blank spectra profile of the different sample cleanup methods.

The separation of the target analyte from a complex matrix is a crucial step before label-free SERS identification. In this work, we first examined the impact of three sample pretreatment techniques (PP, UF, and ms-SPE) for human serum cleanup that were combined with the D-SERS platform. Figure 3 illustrates the average SERS spectra and heat map, representing the intensity distribution of the band at 1559 cm^–1^, for a MER concentration of 750 μM spiked in commercial human serum and separated by each method. Noticeable differences are identified in band spectral complexity, noise background contribution, analyte distribution, and localization on the SERS substrate, all of which have significant impacts on drug identification and sensitivity.

Although employing PP allows one to show the main MER band (1559 cm^–1^), its subsequent spectral identification from the blank in other band regions is restricted. The UF method was performed by using 3 and 10 kDa filter cutoffs (Figures S8 and 3b, respectively).

An improvement in SNR is obtained with the 10 kDa filter, allowing us to distinguish more than one MER characteristic band (1559, 890, and 771 cm^–1^). The higher blank signals observed with the 3 kDa UF filters compared to PP and the 10 kDa UF filter may be attributed to the accumulation of small molecules from the serum that compete with MER for adsorption on the metallic surface.

In contrast, using ms-SPE resulted in the most effective method for serum cleanup, reducing the amount of interfering molecules, which improved the SNR value and band intensity identification with more wavenumbers (1559, 1387, 1059, 890, and 771 cm^–1^). For instance, the uric acid band intensity (640 cm^–1^) is prominent with the UF and PP methods. However, this band is of low intensity with the ms-SPE method, reflecting its efficiency for removing both large and small molecules from the serum. Therefore, the optimal balance between separation and spectral identification was achieved by performing the ms-SPE method.

### Chemometrics Analysis on Instrumental Performance

The performance of our developed D-SERS platform was compared to a commercial Raman spectrometer, which was used together with a spin-stand module for sample centrifugation on the disc. Serum samples spiked with different MER concentrations were pretreated following the ms-SPE method, and analytes were measured in parallel with the commercial Raman system and the D-SERS device.

**Figure 4:**
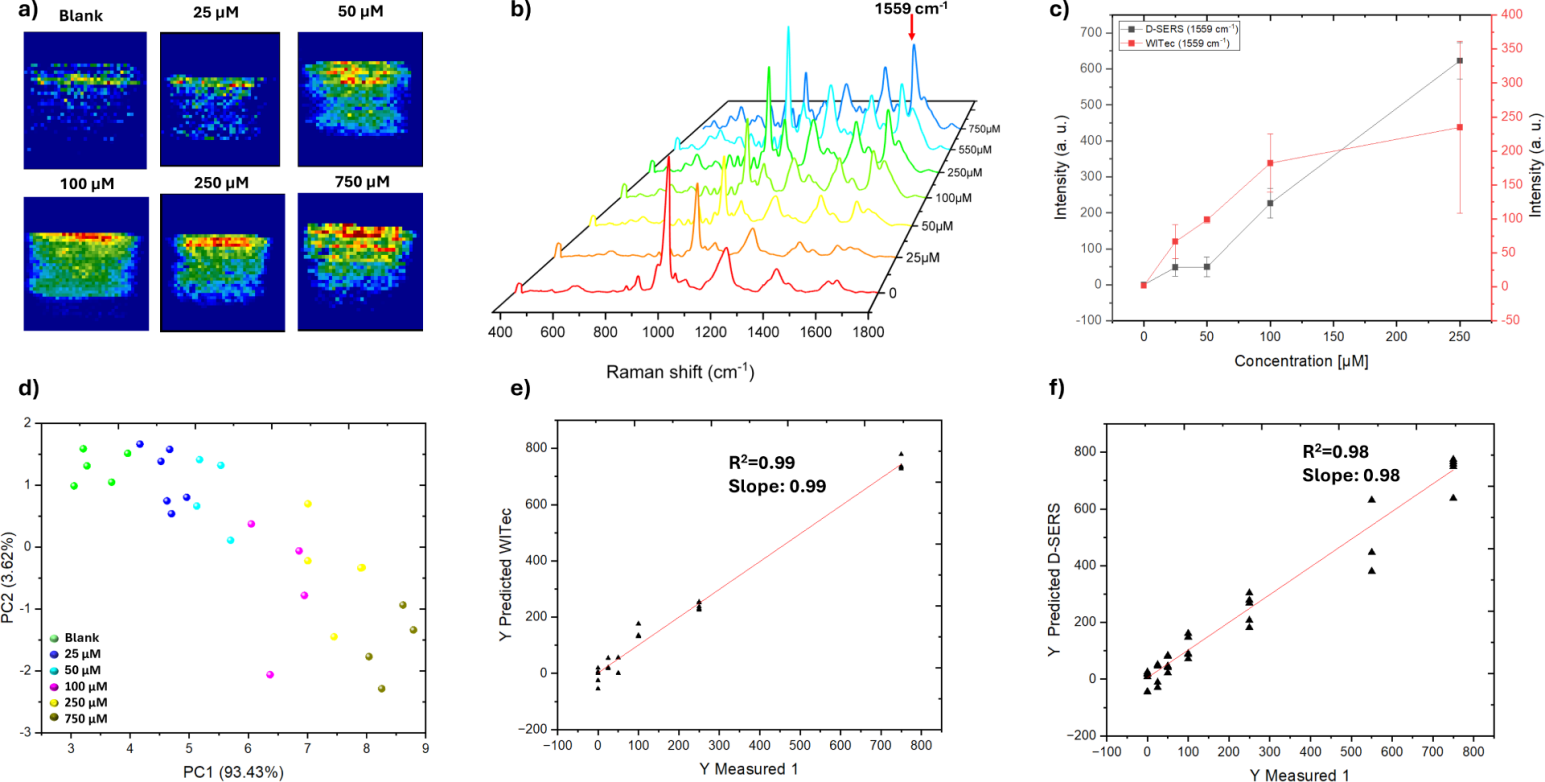
(a) Heat maps of the SERS chips for different concentrations based on the prominent signal of MER at 1559 cm^–1^. (b) 3D overlaid spectra of MER in different concentrations with D-SERS. (c) Univariate concentration-dependent calibration curves, comparing both systems, employing the band at 1559 cm^–1^; all concentrations were measured five times (*n* = 5 ± SD, error bars). (d) Score plot of PC1 vs PC2 on the concentration-dependent data set obtained from D-SERS. PLSR model on the data sets of (e) commercial Raman system and (f) D-SERS.

Figure 4a,b shows the heat maps of the 1559 cm^–1^ band intensity distribution and the 3D overlaid plot of the concentration-dependent spectra, respectively. The main band intensity shows an increase proportional to the MER concentration. The performance of both devices was compared by building independent univariate calibration curves employing the band intensity at 1559 cm^–1^ with optimized parameters (Figure 4c).

A good correlation is obtained by employing both systems. Although the commercial system performs better for MER detection at low concentrations (25, 50, and 100 μM), the obtained SD values are higher than those of the D-SERS system.

To improve the robustness of MER quantification from both Raman systems, we implemented multivariate approaches rather than just observing one band intensity dependence. First, we performed PCA analysis to simplify the data and reveal the spectral variables that are shared among the samples. The implemented PCA with data from the D-SERS system contained 30 rows and 696 Raman shifts. The RMSE analysis of calibration and cross-validation shows that there are 6 PCs that contribute to separating and classifying different sample concentrations. Figure 4d shows the plot of the two most important PCs (PC1 and PC2) against each other, showing a clear concentration-dependent clustering pattern.

Figure S9 shows the plot of the obtained loadings (components against variables), showing the impact contribution to the PCA classification, where bands at 1559 and 889 cm^–1^ contribute the most, making the differences between these clusters. This suggests that MER identification can employ more than one characteristic band. However, implementing a multivariate method could provide better accuracy for a quantitative label-free SERS approach.^15^

Furthermore, for the robustness of MER quantification with a multivariate approach, we implemented a PLSR algorithm with custom-made and PLS-toolbox software. To enhance the model’s accuracy, various preprocessing steps, such as autoscaling, mean centering, normalization, derivatives, and logarithms, were applied to the concentration-dependent data sets. The obtained *R*-squared values for calibration and cross-validation after each method are compared in Table S2. It was determined that the min-max scaling preprocessing method was the most effective, as it standardizes the range of values for each feature within a data set to fall between 0 and 1. This normalization assists in equalizing the influence of different features by placing them on a comparable scale.^31^ In the case of SERS data analysis, where Raman shifts are crucial for distinguishing between clusters, min-max scaling can help to ensure that the prominent distinctive bands, such as those at 1559 and 890 cm^–1^, receive appropriate emphasis in the model.

Following this approach, the performance for MER quantification was evaluated with the PLSR models in a D-SERS device and a commercial Raman system. The data sets from the commercial Raman system to build the matrix consisted of 30 rows and 1024 Raman shift values, while D-SERS data sets were the same as the data set in which PCA was implemented. Table 1 shows the obtained values for each system based on precision (SEP), accuracy (bias and RMSEP), sensitivity, and analytical sensitivity. These values show that the D-SERS displayed good precision and accuracy, with SEP_D-SERS = 53.52 μM, bias_D-SERS = 0.32 μM, and RMSEP_D-SERS = 53.46 μM. On the other hand, the commercial Raman microscope also exhibited commendable precision and accuracy values, with SEP = 86.12 μM, bias = 2.21 μM, and RMSEP = 85.1 μM.

**Table 1:** Figures of Merit for PLSR Model Obtained with D-SERS and the Commercial Raman System

Figure of merit and fitting	Parameter	D-SERS	Commercial Raman
Accuracy	RMSEP	53.46 μM	85.1 μM
Systematic error	BIAS	0.32 μM	2.21 μM
Precision	SEP	53.52 μM	86.12 μM
Sensitivity	SEN	3.44	4.05
Analytical sensitivity	γ	0.31 μM^–1^	0.19 μM^–1^
Inverse of analytical sensitivity	γ^–1^	3.22 μM	5.26 μM
LoD	-	12.12 μM	23.31 μM
LoQ	-	36.37 μM	47.54 μM
Fitting	*R*^2^ calibration	0.95	0.91
	*R*^2^ cross-validation	0.88	0.88
	*R*^2^ prediction	0.93	0.86

Both systems showed comparable analytical sensitivity, suggesting similar capabilities for MER detection and quantification in commercial human serum after ms-SPE pretreatment. However, the D-SERS system shows improved SNR spectral values, as it contains a larger laser spot size of 70 μm compared to 18 μm in the commercial Raman system. A larger spot size allows a large area of Raman scanning on the highly uniform Ag substrate, reducing the risk of missing information from the analyte distribution on the SERS substrate and obtaining more representative pixel spectra.^32^ Despite similarities in sensitivity values, the D-SERS system provides users with practical advantages of being small, compact, light, and easy to operate, which is highly beneficial in hospitals and clinical settings.

### Assay Validation with Clinical Samples from ICU

To demonstrate the applicability of our developed method for MER quantification in clinical samples, we analyzed 27 heterogeneous ICU patient serum samples, collected at different time points following initial MER administration. The MER quantification was performed both in the D-SERS device and in HPLC, as the reference method. The obtained MER quantitative values from each method are detailed in Table S3.

The MER quantification in patient samples with PLSR was performed using two approaches. In the first approach, the PLSR model was built with a calibration data set employing commercial serum samples. The second approach used PLSR models that were built with real patient serum data, employing HPLC results.

For the second approach, 12 PLSR models were built. In each iteration, two or three samples were excluded as prediction data sets, and the rest of the patient samples were used as calibration data sets. To avoid overfitting of the model, parameters like root-mean-square error (RMSE), cross-validation, and prediction were evaluated (Figure S10).

Furthermore, a permutation test was conducted to validate the robustness of the model (Figure S11). Scatter plots, comparing measured vs predicted MER concentrations, and Bland–Altman plots for assessing the analytical agreement were generated for both approaches to evaluate the performance of the models.

**Figure 5:**
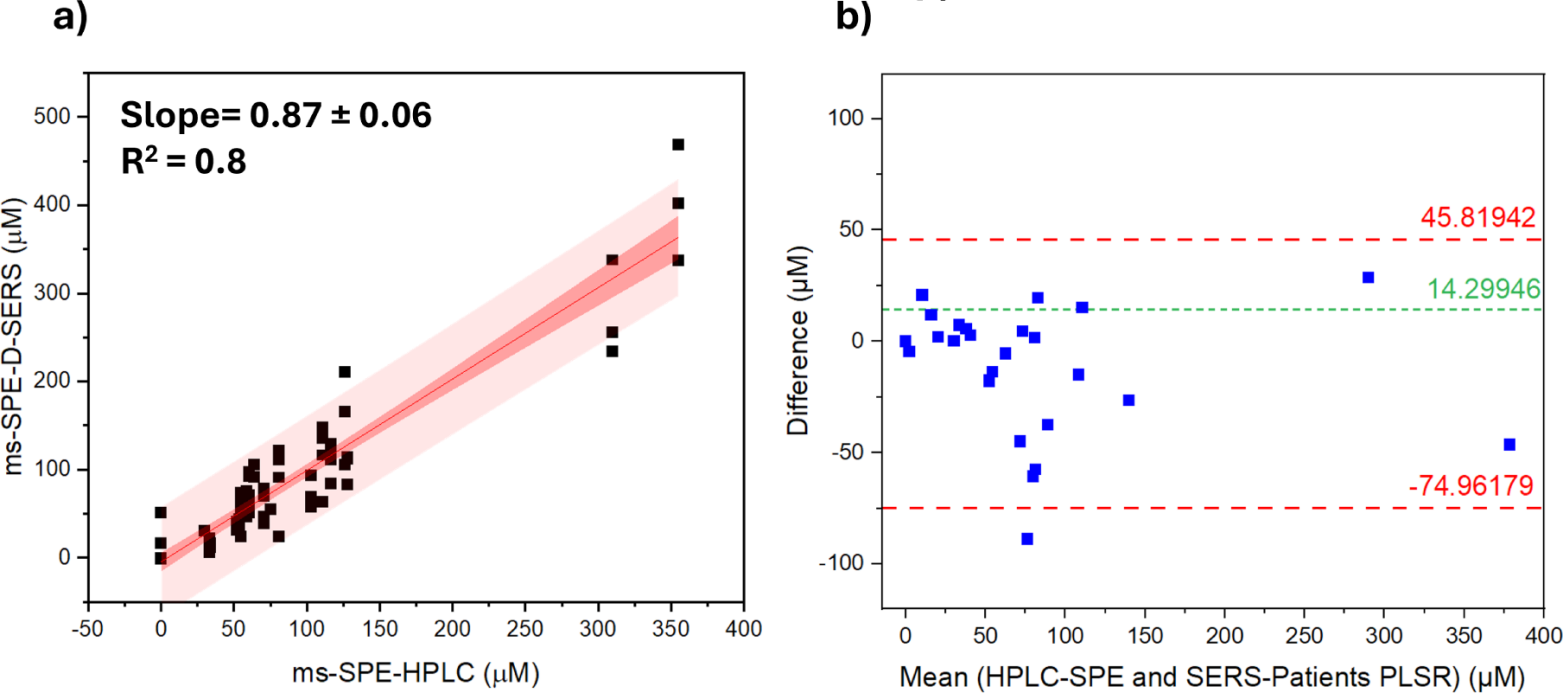
(a) Scatter plot built using SPE-HPLC as the reference method and the D-SERS method combined with PLSR as the test method on the patient sample data set. The red line represents the regression line, while the 95% confidence band is depicted as a red area. (b) Bland–Altman plot built using HPLC with SPE as the reference method, and the developed SERS method combined with PLSR multivariate data analysis as the test method. The short-dashed green and dashed red lines represent bias and 95% limits of agreement, respectively.

The scatter plots of both approaches (Figures 5a and S12) showed a more accurate prediction for the PLSR model based on patient samples (*R*^2^ = 0.8) than the commercial serum approach (*R*^2^ = 0.68), with most of the samples within a 95% confidence interval. Indeed, employing the spectral patient data for the calibration tends to reflect more accurately the inherent sample variations in composition that might also influence the prediction.^33^ The Bland–Altman plots (Figures 5b andS13) provided an overview of the agreement between the predicted and measured MER concentration. The patient-based approach demonstrates a narrower range of agreement with an upper limit of agreement (ULA) of 45.8 μM and a lower limit of agreement (LLA) of −74.9 μM, while for the commercial serum approach, the ULA and LLA were calculated to be 119.6 and −94.1 μM, respectively (Figure S13).

The tighter agreement range demonstrates predictions’ improved reliability and consistency when real patient serum data are used for the calibration.^34^ The values obtained with D-SERS for the MER quantification showed a 14.3 μM bias in comparison with results from HPLC. All of the samples, except one, are located within the range of 95% limits of agreement (LoA).

The analytical comparison of our device against HPLC showed good performance for MER quantification in patient samples. The clinical significance of the obtained bias value from the Bland–Altman analysis was evaluated based on established MER therapeutic ranges. The minimal detectable change (MDC), calculated from our data, is 60.38 μM (23.15 mg/L), which is just below the reported toxicity threshold of 24 mg/L.^35^ The bias value and 95% LoA indicate some variability, limiting instrumental interchangeability at this stage. However, our ongoing efforts to improve those values are focused on refining the method and exploring potential factors influencing discrepancies. This involves incorporating more clinical samples and additional patient data, such as age, weight, gender, and experimental conditions. This approach aims to improve agreement and further validate the method for clinical application.

Nevertheless, there are several factors that might contribute to the discrepancy in the accuracy of our quantification results. For example, the patient-to-patient sample complexity and inherent physiological variations, as samples, were provided from a heterogeneous group of anonymous ICU patients receiving MER in routine care. Thus, no clinical data were collected. Interpatient variability might also impact the MER metabolism and pharmacokinetics under the treatment. Moreover, ICU patients usually present with different comorbidities and physiological variations that impact the blood properties and metabolite composition.^36^ Additionally, ICU patients in the hospital receive other medications, which might include antiarrhythmics, anticoagulants, sedatives, and other antibiotics.^36^ The presence of several small molecules in the sample can also impact the label-free SERS detection and quantification, as they can compete with the MER on the plasmonic metallic surface.

To the best of our knowledge, this is the first study involving a SERS assay or a sensor-based method for MER detection and quantification in a biological sample. In comparison to conventional methods (Table S4) such as HPLC, LC-MS, and other less commonly used techniques like the electrochemical method,^37^ microbiological assay (MBA),^38^ capillary electrophoresis (CE),^39^ and micellar electrokinetic chromatography (MEKC),^40^ our developed SERS-based method for quantifying MER offers several significant advantages.^41^

The D-SERS assay demonstrates high performance, sensitivity, and specificity for MER quantification, fitting the suggested therapeutic range (20–450 μM) in clinics,^7^ and results are obtained within minutes. It is obvious that implementing a sample pretreatment method, such as SPE, involves extra time in sampling and handling and requires technical expertise, making it less feasible for untrained personnel in clinical settings. However, in label-free SERS approaches, it is important to employ a sample pretreatment method to reduce serum complexity, and SPE is one of the most efficient, which requires a low volume of sample and allows analyte up-concentration. Moreover, several recent works have shown novel compact setups, like robotic systems, facilitating SPE automation and its coupling to almost any analytical setup.^42,43^ These systems reduce time and variabilities, minimize human intervention, and make the method feasible for clinical high-throughput. Therefore, we do not expect that the performance of the SPE in clinical settings can be a limitation for our device application. Yet, it is true that aiming for the development of devices that contain a sample pretreatment and sensing module is crucial for providing a fast answer.

Despite the fact that the SPE sample preparation step in our assay involves around 15 min, a total MER quantification can take around 20 min. Moreover, the device provides a competitive advantage in terms of versatility and cost, as it lowers the use of reagents and avoids the need for surface functionalization. Multiple analyses can be performed in the same cartridge as eight samples can be measured simultaneously within 5 min of full chip scanning per sample. Therefore, up to eight different patient samples can be analyzed in less than 1 h. Moreover, our D-SERS device represents a significant advancement in chemical analysis by integrating a Raman microscope, a spindle motor, and a versatile microfluidic setting in a compact measuring device [∼ 5 kg and 25 × 35 × 30 cm], which is easy to operate and can be placed in ICU settings close to the patient.

## Conclusions

In summary, in this work, we developed, for the first time, a compact SERS device (D-SERS) and assay for rapid label-free quantification of meropenem in ICU patient’s serum samples. The custom-made and compact D-SERS device consisted of a microfluidic cartridge with integrated nanopillar SERS substrates, a spin motor, and a Raman spectrometer, equipped with software for scanning and multivariate data analysis. The combination of the D-SERS device with ms-SPE, as a sample pretreatment, allowed for sensitive label-free MER detection, satisfying the meropenem clinical therapeutic window. We obtained a good correlation by comparing our D-SERS device with a commercial Raman system, highlighting its efficiency and reliability in label-free analysis. Furthermore, our assay and the chemometric data analysis method for quantification were validated with clinical serum samples from ICUs, against HPLC, showing good agreement. Although the sample pretreatment involves extra time analysis, a total MER quantification is performed within 20 min, significantly shortening the time employed with conventional analytical settings in the ICU. Therefore, our work shows the benefits of developing compact and easy-to-use devices for fast and accurate antibiotic quantification in clinics, advancing the point-of-care in drug monitoring.

## Supporting Information Available

Experimental details, data analysis, and methods, along with photographs of the disc design and software interface; furthermore, it provides a table of the actual concentration of meropenem in real patient samples, Raman shifts of different functional groups, and a comparison with previous works (PDF)
