# Supplementary material for: Improving Meropenem Quantification in a Compact SERS-Based Centrifugal Microfluidic Platform: Toward TDM of Antibiotics in ICU
Source: Anal Chem. 2025 Mar 25;97(14):7888–96. doi: 10.1021/acs.analchem.4c06902 (PMC12004356; doi:10.1021/acs.analchem.4c06902)
Supplement: Supplementary file 1 — ac4c06902_si_001.pdf [file ac4c06902_si_001.pdf]

Supporting Information

Improving meropenem quantification in a compact SERS-based centrifugal microfluidics platform: Towards TDM of antibiotics in ICU

Martyna A. Pytlarz[1], Gohar Soufi[1*]‡, Isidro Badillo-Ramírez[1*]‡, Laura Serioli[1], Roman Slipets, Anders Perner[2] & Anja Boisen[1]

1 Center for Intelligent Drug Delivery and Sensing Using Microcontainers and Nanomechanics (IDUN),

Department of Health Technology, Technical University of Denmark, Kongens Lyngby 2800, Denmark.

2 Department of Intensive Care, Copenhagen University Hospital – Rigshospitalet, Copenhagen 2100, Denmark.

3 Department of Clinical Medicine, University of Copenhagen, Copenhagen 2200, Denmark.

Table of Contents:

M-S1. Chemicals and materials

M-S2. SERS substrate fabrication

M-S3. Design and fabrication of microfluidic discs

M-S4. Compact Raman spectrometer with incorporated spin motor.

M-S5. Raman and SERS characterization with commercial Raman spectrometer.

M-S6. Sample pre-treatment.

M-S7. NPAS method on the disc

M-S8. HPLC reference method

Figure S1. Centrifugal microfluidics design

Figure S2. Schematic of the portable Raman machine

Figure S3. Schematic of the ms-SPE procedure

Figure S4. SERS characterization of MER employing an Au-based SERS NP substrate.

Figure S5. Control software interface displaying the programming for spinning speed in centrifugal microfluidics.

Figure S6. The data analysis software's interface with different options

Figure S7. The data analysis software's interface for multivariate regression with the capability of making models and back calculations.

Figure S8. Overlaid spectra of 750 μM of Meropenem in serum sample and blank after ultrafiltration with 3 kDa cutoff.

Figure S9. Loading plot of PC1 and PC2 on

Figure S10. Root mean square of error for calibration, cross-validation and prediction

Figure S11. Histogram of R2 values obtained from the permutation test for the PLSR model.

Figure S12. The scatter plot of the predicted concentration from D-SERS vs measured concentration from HPLC by the PLSR model based on the commercial serum calibration with 4LVS.

Figure S13. Bland-Altman plot built using HPLC with SPE as the reference method

Table S1. Raman and SERS band assignments

Table S2. Different preprocessing before PLSR on the concentration-dependent data sets of MER obtained with D-SERS

Table S3. MER quantitative values in clinical serum from ICU, patient samples, obtained with HPLC, as the reference method, and the ms-SPE to D-SERS assay.

Table S4. Comparison of different reported methods for MER quantification

Methods

M-S1. Chemicals and materials. Meropenem (certified reference material), commercial human serum (human male AB plasma), methanol (≥99.9% purity) and PBS, pH 7.4, were purchased from Sigma Aldrich (St. Luis, MO, USA). A meropenem stock solution (10mM) was prepared in ultrapure water (Millipore Corporation, Billerica, MA, USA). A fresh stock solution was prepared before each measurement to prevent drug degradation. Meropenem working solutions were prepared by diluting the stock solution in commercially available human serum to desired concentrations (25-750 μM).

Samples pretreatment was conducted using 0.5 mL centrifugal filter units, with a pore size 3 and 10 kDa (Amicon Ultra, Merck Millipore Ltd.IRL) for ultrafiltration, and commercially available solid phase extraction C18 columns (MonoSpin L, GL Sciences).

M-S2. SERS substrate fabrication. The silver and gold plasmonic NP structures were fabricated in two main steps: etching and metal deposition. First, NP structures were obtained on silicon wafer using maskless reactive ion etching (RIE) using Advanced Silicon Etcher. RIE was conducted with SF_6/O_2 process gasses over 6-inch wafer for 4 minutes, yielding NP of ~400nm in height and with

a density of ~20NP/µm2. The next step after etching was then O_2 plasma cleaning. SiNP structures were exposed for one minute to eliminate sulphur-fluoride based etching by products (MESC Multiplex ICP, Surface Technology System, Morgan Hill, CA, USA). In the last step, the plasmonic property of SiNP was obtained by metal deposition. Nanopillars were coated using a conventional deposition technique (e.g. electron-beam evaporation) by 225 nm thick gold or silver metal film. In consequence, “grass-like” structures with round caps on top of pillars were obtained.

The obtained wafers were characterized using Scanning Electron Microscopy (SEM) (Zeiss Supra VP 40, Jena, Germany). The produced wafers were diced from the back side using a Laser Micromachining tool (3D-Micromac AG, D-09126 Chemnitz, Germany) resulting in 4x4 mm chips. Before and between use, the chips were stored under vacuum.

M-S3. Design and fabrication of microfluidic discs. Microfluidics disc design and optimization were performed using computer modeling techniques as previously reported by Serioli et al. [unpublished manuscript]. Discs design consisted of eight units per disc, in which each unit consisted of 4 chambers including two loading chambers, a mixing chamber, and a sensing chamber, where the nanopillar-based SERS substrate is placed [better to add an image-scheme of the disc]. Discs are produced by gradual assembly of laser-cut poly(methyl methacrylate) (PMMA) and pressure-sensitive adhesive layer (PSA) (ARcare 90106, Adhesive Research, Limerick, Ireland). Three layers of PMMA are cut using an ablation laser machine (Epilog Laser, Houten, The Netherlands). The top and bottom layers are 0.5 mm thick, and the middle layer with inner wells is 1 mm thick. PSA is cut with precision blade cutter (CE-40, Graphtec Corp., Yokohama, Japan). The disc is assembled step by step and bonded with 4 kN.

M-S4. Compact Raman spectrometer with incorporated spin motor. The compact Raman device (**Figure S2**) was built at home, consisting of a compact spectrometer module (Wasatch Photonics, USA), covering a wavenumber range of 200 - 2100 cm^{-1}, a 25 µm slit with 8 cm^{-1} spectral resolution, a single mode laser at 785 nm wavelength, and standard sampling optics for approximately 70 µm laser spot size on the sample. It also contains a motorized XYZ stage, based on 8CMA06 (Standa Ltd, Lithuania), providing a scanning volume of 13 x 13 x 13 mm^3; a centrifuge module with a brushless spindle motor, DC motor EC-i 40 Ø40 mm, 50 Watt, EPOS4 Compact 50/5 CAN; and a digital positioning controller (Maxon International Ltd., Switzerland), capable of rotation speeds up to 7200 rpm. A full schematic representation of the Raman device is shown in Figure X. The software for controlling and analyzing data was developed using Delphi RAD Studio (Embarcadero Technologies, USA), and Python (Python Software Foundation).

M-S5. **Raman and SERS characterization with commercial Raman spectrometer**. Raman and SERS scanning analysis were performed in a commercial instrument with WITec Alpha 300R microscope (Oxford Instruments), equipped with a 785 nm wavelength laser excitation. For Raman analysis, the sample was visualized with 10x. Large map scanning was performed for SERS measurements by visualizing the entire chip with a 10x objective (Zeiss – NAME), using 0.05 s integration time for each step (50 points per line and 50 lines per image). The power of the laser

was set to 10, 20 and 50 mW for SERS mappings, disc measurements and spontaneous Raman, respectively.

M-S6. Sample pre-treatment. For analysis of Meropenem, a pretreatment step was required to clean-up the samples before SERS measurement. Three common methods were implemented: protein precipitation, ultrafiltration, and spin column solid phase extraction (ms-SPE). An additional sample purification step was achieved by passing the sample through NP substrate (NPAS procedure). While the sample was moving on the substrate surface, small particles were separated from larger ones that could be expected in serum (e.g. proteins). The bigger components stayed at the bottom of the chip as they were filtrated by grass- like structures, and particles of interest moved up to form hot spots.

i) Protein precipitation. When adding organic solvent to human serum, precipitation occurs. Both serum and solvent were applied directly on the disc, where they were mixed in 1/5 ratio. Biological components aggregated and sediment was pushed through microchannel to sensing chamber.

ii) Ultrafiltration. Serum-meropenem samples were ultrafiltered before mixing with the organic solvent (1/5 ratio). Before utilizing them, filters were rinsed with PBS pH 7,4 (10 000 rpm, 30 min). Then, 500 µl of the sample was loaded and centrifuged at 10 000 rpm for 30 minutes. Transparent filtrated solutions were used for NPAS procedure on the disc.

iii) Mono-spin solid phase extraction (ms-SPE). The technique was utilizing SPE C18 columns. The ms-SPE procedure is divided into following parts: (i) Conditioning: after the column filter was inserted into a centrifuge tube, it was conditioned in two steps, with MeOH and water (500 µM). Both times it was centrifuged for 2 min at 2000 rpm. (ii) Loading: serum-meropenem solution in desired concentration was diluted with water (1,5 ml sample with 3ml of water), loaded to the column and centrifuged at 1000 rpm during 7 min. (iii) Washing: 500 µL of water was centrifuged in column for 1 min at 4000 rpm. (iv) Elution: 300 µL of MeOH and water (2/1 ratio) was added to column and centrifuged 1000 rpm during 2 min. Collected sample was placed again (three times in total) into the column and centrifuged with the same centrifugation conditions.

Eluted solutions were collected and analyzed with NPAS procedure, on the disc, where the sample was injected directly to the mixing chamber and thus directly pushed through microchannel to the substrate (no mixing step with additional solvent, since as the elution process was done with methanol, there was no need of adding additional wetting agent before measurement).

M-S7. Centrifugal conditions on the disc. On the disc, 13 µL of serum-meropenem sample was introduced with micropipette to the small loading chamber. Similarly, 65 µL of methanol was injected into the large loading chamber. Secondly, the disc was spined with a controlled software interface (**Figure S4**) with a frequency of 20 Hz and acceleration 10 Hz/s to transfer both sample and solvent to the mixing chamber. Then the rotation was stopped. In the next step, both fluids were mixed by adjusting acceleration to 75 Hz/s together with frequency 10 Hz, while the mixing amplitude was equal to 30 degrees. These settings were forcing rotating platform to move

clockwise and anticlockwise and the fluid inside mixing chamber was being shaken. The shaking was done for one minute and stopped. After that, acceleration was put back to 10 Hz/s and frequency was increased gradually from 0 to ~70 Hz to push the liquid from the mixing chamber through microchannel to the substrates that were attached to the bottom of the sensing chamber. In detail, once the liquid level touched the chip (~35 Hz), the frequency was increased by 5 Hz every 5 s until 70 Hz. Consequently, the chip was gradually covered with the liquid up to ~75%. Last, the frequency was significantly decreased (to 10 Hz) to remove the sample from the sensing chamber back to the mixing chamber. The disc was then entirely stopped and left to dry to cause nanopillar leaning and "hot spots" formation.

Overall, SERS substrate needs to be wetted to enable liquid movement. As those are hydrophobic, any kind of organic solvent can serve as wetting agent. For the disc measurements, only methanol was compatible with PMMA (cracks and liquid leakage observed for other solvents).

M-S8. HPLC analysis. The HPLC reverse phase method was developed as reference for meropenem quantification in commercial serum and patient samples after ms-SPE. A Shimadzu Prominence HPLC system (Shimadzu, Kyoto, Japan), equipped with an auto-sampler (SIL-20A, Shimadzu, Kyoto, Japan) and a photo-diode array (PDA) detector (SPD-M20A, Shimadzu, Kyoto, Japan) was employed. The stationary phase consisted of a C18 column (2.1 x 250 mm, 5 µm) with a guard column (C18, 4.6 x 12.5 mm, 5 µm, 95 Å 400 bar pressure limit), both from Agilent Technologies (Santa Clara, California, USA). The column was heated at 40°C. The gradient mode was utilized consisting of 15% ACN and 85% PBS as initial condition. The ratio of ACN was increased up to 30% within 15 minutes and decreased to the initial ratio up to 20 min. The injection volume was 30 µL and the peak areas recorded at 254 nm.

M-S9. Data analysis. A minimum of three distinct SERS substrates were measured (five for calibration curves) under identical experimental conditions. Data analysis was performed using custom-made software (SERSanalyser, Dr. Roman Slipets, DTU) (**Figure S5**). Each collected spectrum was cropped, and it was followed by background correction wheel (with wheel radius 100 and elliptic coefficient equal 5). Next, to obtain the mean value of the signal coming from the molecule of interest, the top 20% of data coming from Meropenem specific band (~1559 cm^{-1}) were considered. Peak intensities at ~1559 cm^{-1} were calculated and averaged between the 5 replicates. For statistical data analysis different tools were used: the PLS toolbox from Eigenvector Research in MATLAB (2021b, MathWorks, Natick, MA, USA), MVC1 toolbox, Python-based tools and Jupyter Notebook and OriginPro 2022., Discriminant and quantitative methods used were Principal Component Analysis (PCA) and Partial Least Squares Regression (PLSR), respectively. Model and prediction were calculated obtaining variety of necessary metrics: the correlation coefficient (R^2), root-mean-square error of calibration (RMSEC), cross-validation (RMSECV), and prediction (RMSEP). The leave-one-out was employed as cross-validation method for calibration data set based on commercial serum containing 6 concentration and 4 replication (24 samples) and Venetian blinds was employed as cross-validation method for the patient-based calibration data set with 27 samples and three replications for each (except four patient that we didn't have

replication). So the independent matrixes were 23* 538 and 65*538, for serum calibration and patient based calibration, respectievely. Furthermore to be ensure about the robustness of the calibration model, the permutation test was applied for the PLSR model. The dependent variable (concentrations) was randomly permuted 100 times while keeping the variable matrix unchanged. The observed R^2 value of the original model was compared with mean R^2 value of the permuted models to be checked that is significantly different.

Outliers were checked using Hotelling and Q residuals. Other Figures of merits including LoD, LoQ, sensitivity, and analytical sensitivity were calculated by the MVC1 toolbox.)., and From the latter AFOMs, the limit of detection (LOD) can be calculated. Considering the so-called type I and type II errors, it can be estimated as:

LOD=3.3 $s0$

where $s0$ is the standard error in the concentration of analyte in an analyte-free sample (the coefficient 3.3 corresponds to 0.05 as α and β probabilities for type I and II errors). It should be noticed, the LOD depends on the concentrations of other components in a given sample. In addition, to calculate the LOD value, it is necessary to estimate the standard error in the predicted analyte concentration for a blank sample ($s0$). This can be done by resorting to:

$$\sigma_y = [\mathrm{SEN}^{-2}\sigma_x^2 + h\mathrm{SEN}^{-2}\sigma_x^2 + h\,\sigma_{y,\mathrm{cal}}^2]^{1/2}$$

Where SEN is the sensitivity, σx2 is the variance in instrumental signals, h is the sample leverage, and σ2y is the variance in calibration concentrations. Also, h is the sample leverage can be defined as follows:

$$h = \mathbf{t}^{\mathrm{T}}(\mathbf{T}^{\mathrm{T}}\mathbf{T})^{-1}\mathbf{t}$$

where T is the PLS score matrix of calibration samples.

But replacing h by the blank leverage ($h0$). However, in the multivariate context the blanks are variable, and thus, there will be a range of blank leverages. Consequently, a range of detection limits, from a minimum LOD (LODmin) to a maximum LOD (LODmax), can be computed (ACS Sens. 2020, 5, 580−587):

$$\mathrm{LOD}_{\mathrm{min}} = 3.3[\mathrm{SEN}^{-2}\sigma_x^2(1 + h_{0\mathrm{min}}) + h_{0\mathrm{min}}\sigma_{y,\mathrm{cal}}^2]^{1/2}$$

$$\mathrm{LOD}_{\mathrm{max}} = 3.3[\mathrm{SEN}^{-2}\sigma_x^2(1 + h_{0\mathrm{max}}) + h_{0\mathrm{max}}\sigma_{y,\mathrm{cal}}^2]^{1/2}$$

Figures:

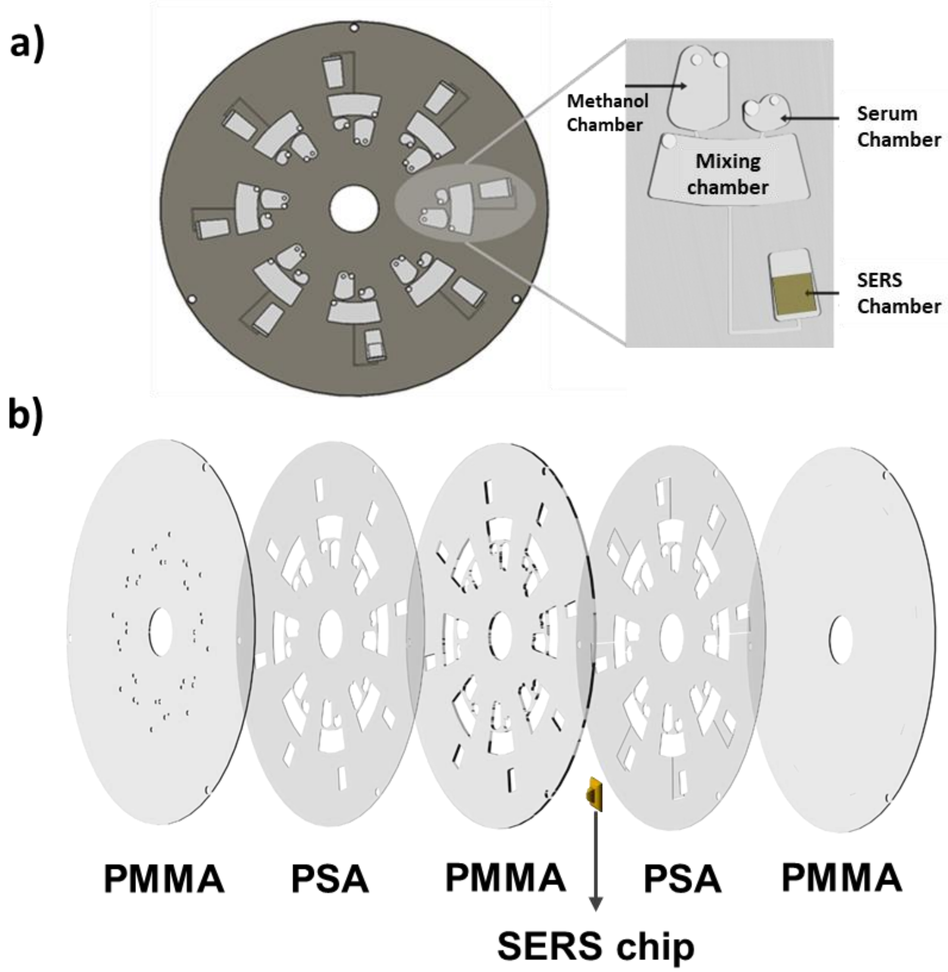

Figure S1. Centrifugal microfluidics design for NPSA-on-disc. (a) Front view of the design and close up to a working unit and its specifications. (b) Explosion view of the disc with different layers.

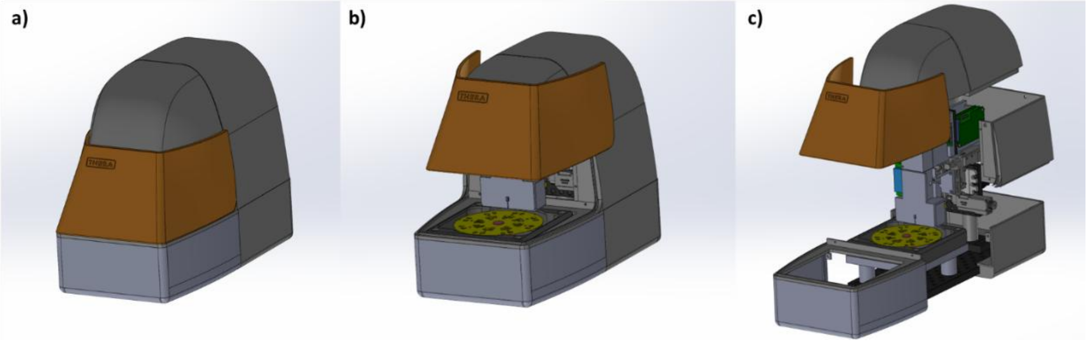

Figure S2. Schematic of the portable Raman machine and of the centrifugal microfluidic cartridge. a) Portable Raman analyzer design with b) open lid and d d) disassembled design with observable spin stand and spectrometer.

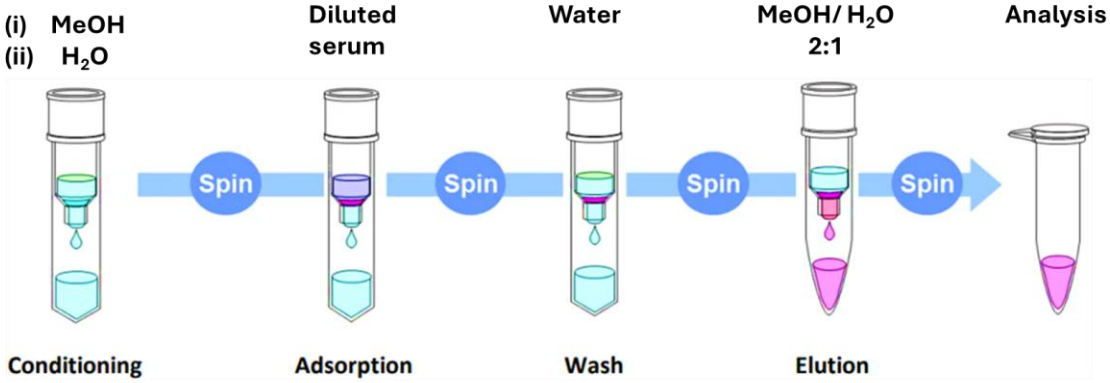

Figure S3. Schematic of the ms-SPE procedure containg four steps: (i) conditioning, (ii) adsorption or loading the samples (iii) washing (iv) elution.

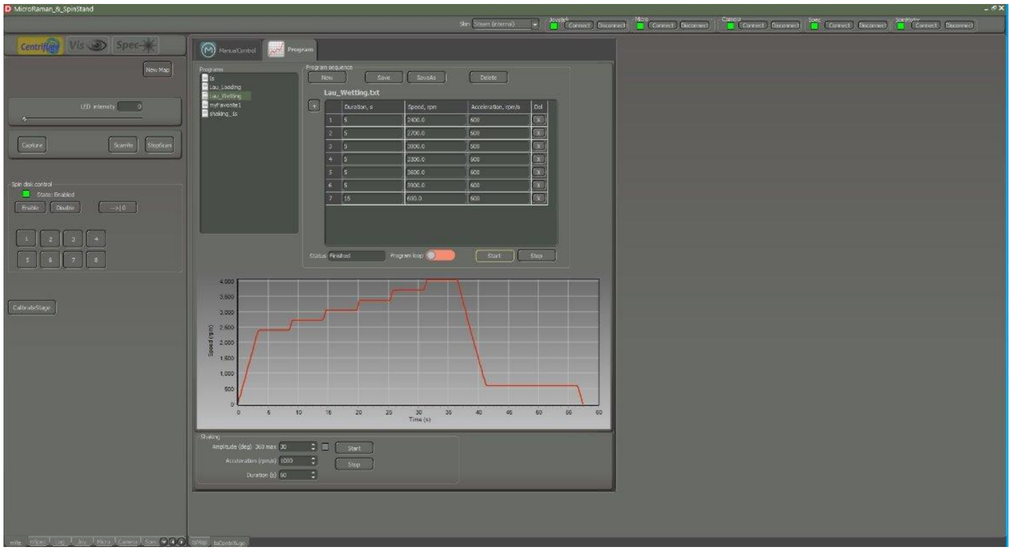

Figure S4. Control software interface displaying automated step-by-step programming to perform the spinning in the centrifugal microfluidics cartridge.

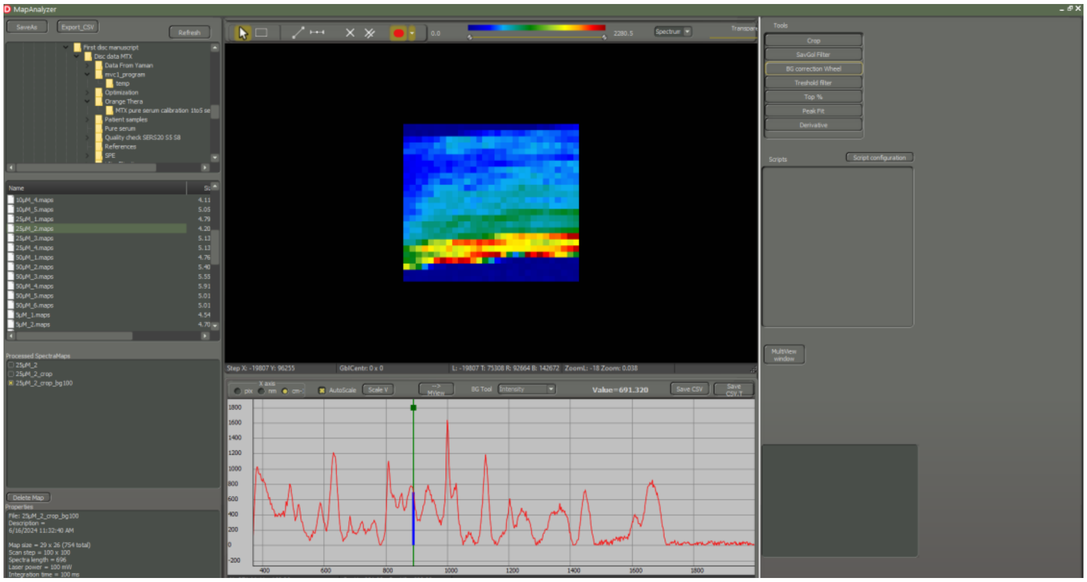

Figure S5. The data analysis software's interface with different spectral data preprocessing options including cropping, background correction, smoothing, average, etc.

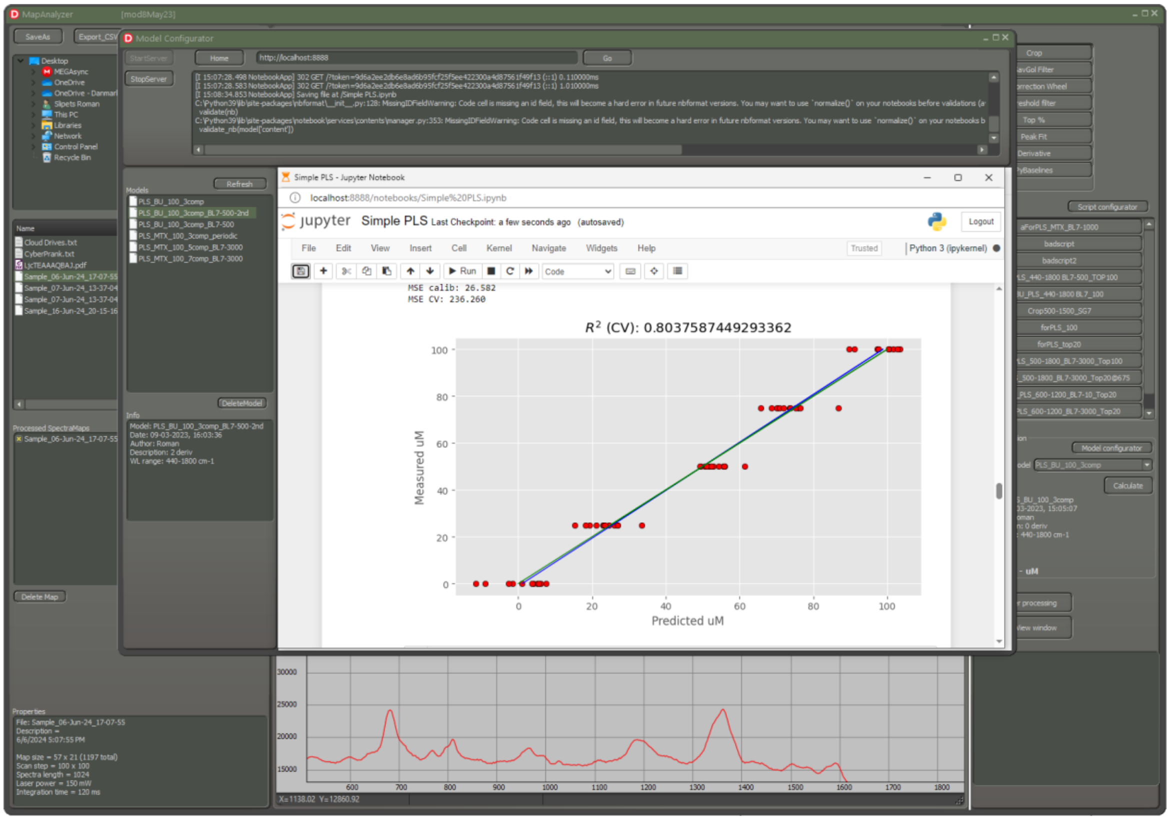

Figure S6. The data analysis software's interface for multivariate regression with the capability of making models and back calculations.

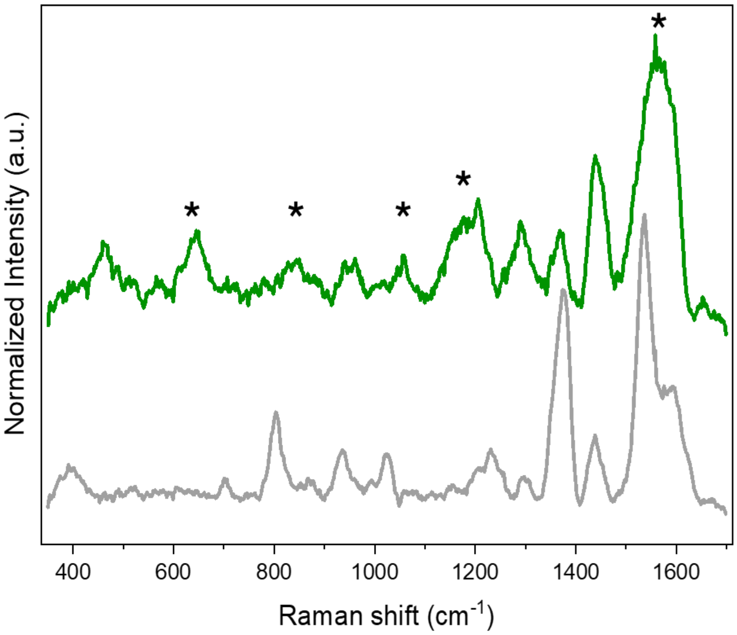

Figure S7. SERS characterization of MER employing an Au-based SERS NP substrate. The green line is the obtained spectrum, grey is the Au reference spectrum, and the indicated bands are those corresponding to MER.

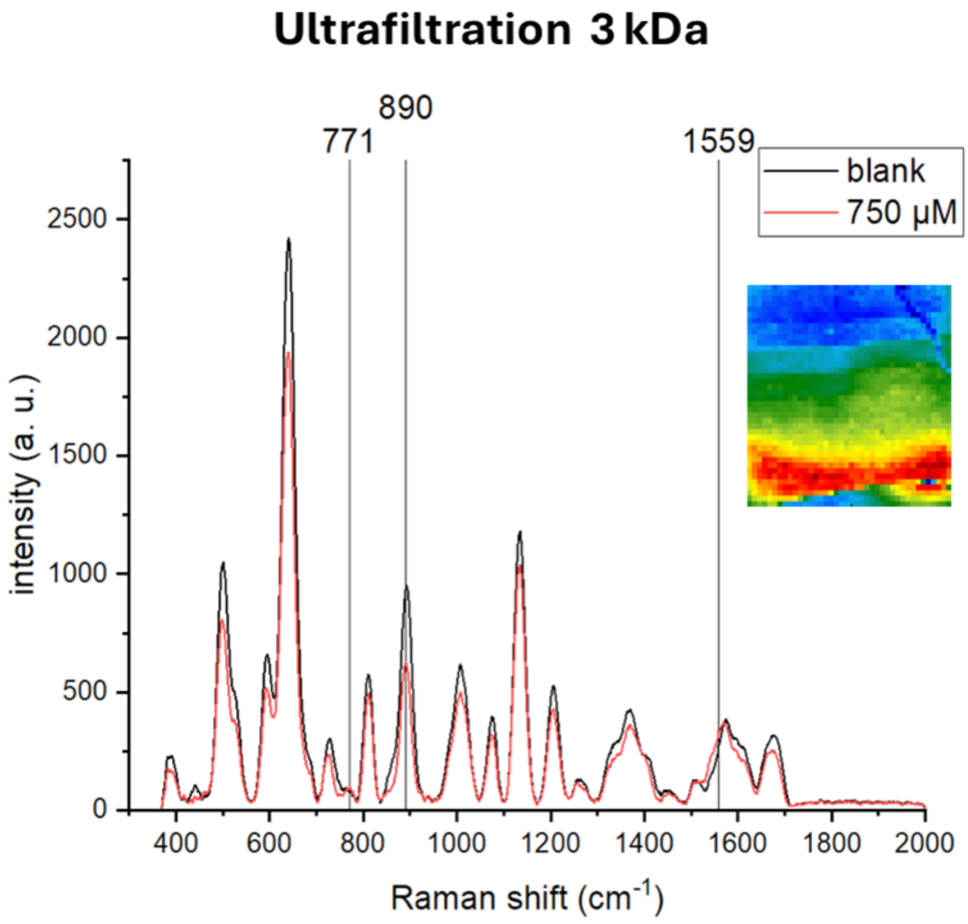

Figure S8. Overlaid spectra of 750 μM of Meropenem in serum sample and blank after ultrafiltration with 3 kDa cutoff.

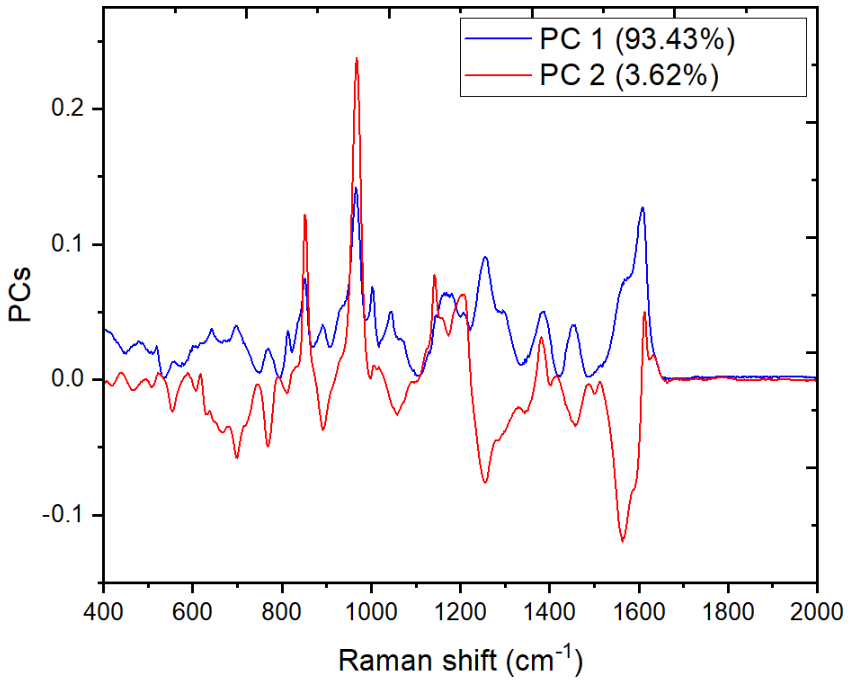

Figure S9. Loading plot of PC1 and PC2 on the concentration-dependent data set obtained from D-SERS.

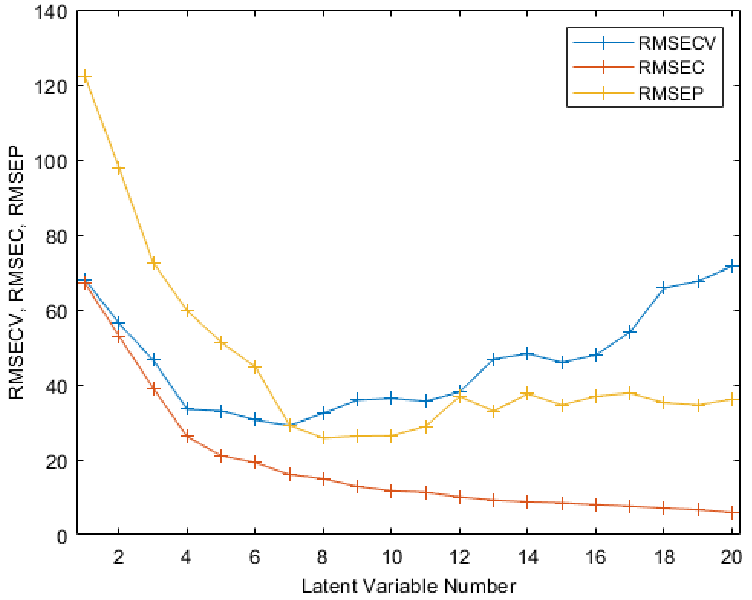

Figure S10. Root mean square of error for calibration, cross-validation and prediction on the Patient sample-based calibration with 4LVs and with Venetian blind cross-validation method.

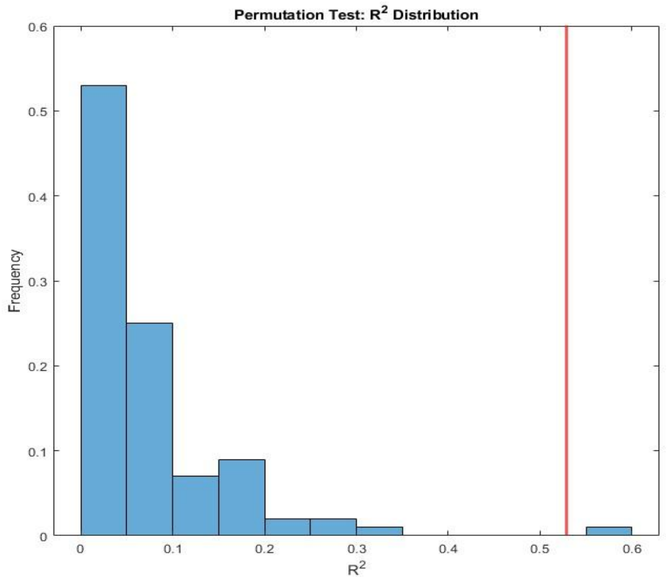

Figure S11. Histogram of R2 values obtained from the permutation test for the PLSR model. The dependent variable (concentrations) was randomly permuted 100 times while keeping the variable matrix unchanged. The observed R^2 value of the original model (0.85) is indicaing that by the red line, significantly higher than the mean R^2 value of the permuted models (0.53).

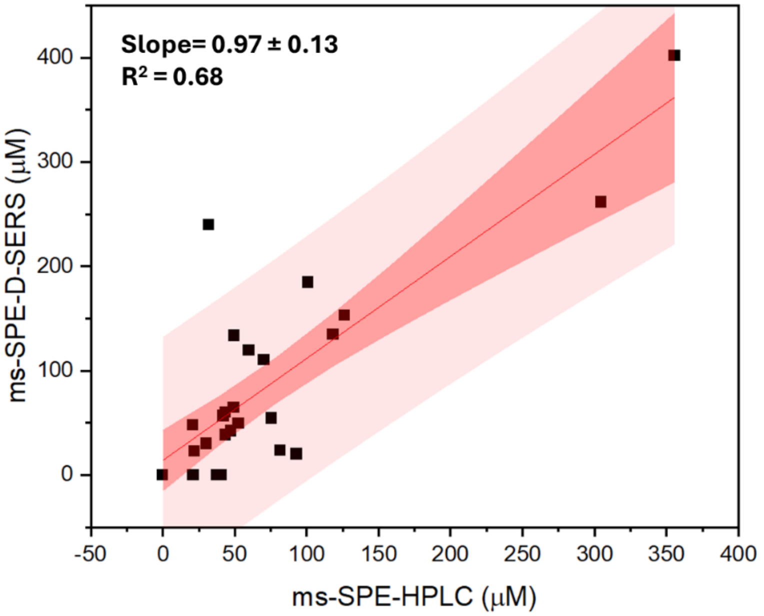

Figure S12. The scatter plot of the predicted concentration from D-SERS vs measured concentration from HPLC by the PLSR model based on the commercial serum calibration with 4LVS.

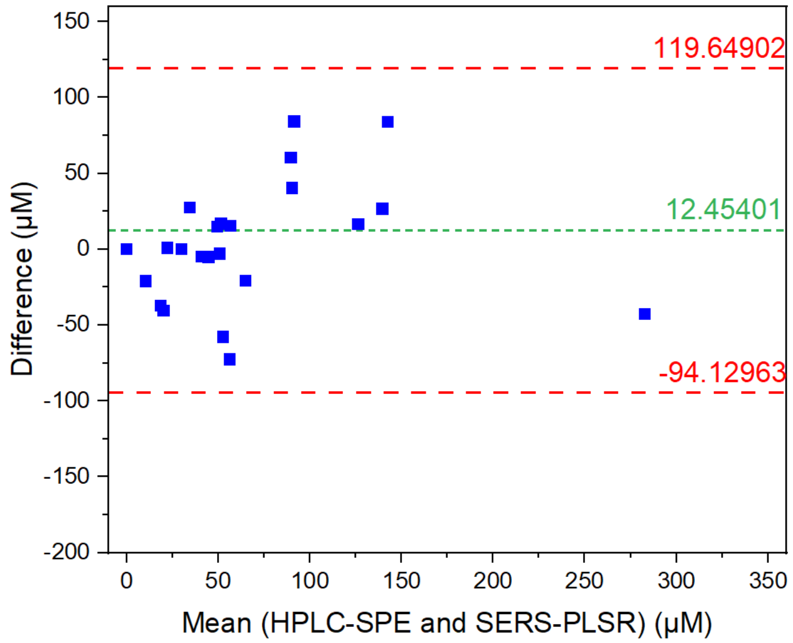

Figure S13. Bland-Altman plot built using HPLC with SPE as the reference method, and the developed SERS method combined with PLSR multivariate data analysis on the commercial serum calibration dataset as test method. The short-dashed green and dashed red lines represent the mean (bias), and 95% limits of agreement, respectively.

Tables:

Table S1. Raman and SERS band assignments of MER

Raman (cm^{-1})	*SERS (in PBS) (cm^{-1})*	*SERS (in serum) (cm^{-1})*	*Band Assignment*
670	668	665	Pyrrolidine ring def.
709			Breathing of β-lactam ring
805	805	771	Breathing of β-lactam ring + C-N str.
894	888	888	C-N str. in β-lactam ring
1052	1061	1066	C-C str. in β-lactam ring
1260	1254		C-N + C-H str. + CH_3 def.
1384	1394	1386	C-N str. in CO and CH_3 domain
1447	1456	1453	CH and CH_2 def.
1550	1558	1558	C=C str. in pyrrolidine ring+β-lactam ring

Str. Stretching and def. Deformation

Table S2. Different preprocessing before PLSR on the concentration-dependent data sets of MER obtained with D-SERS

NO.	PREPROCESSING	R^2 CALIBRATION	R^2 CROSS-VALIDATION
**1**	none	0.96	0.71
**2**	Autoscale	0.99	0.55
**3**	GLog & log10	0.92	0.71
**4**	Derivative	0.98	0.55
**5**	Reference/Background correction	0.98	0.58
**6**	Smoothing	0.97	0.57
**7**	SNV	0.89	0.53
**9**	Variable Alignment	0.98	0.58
**10**	Variance Std Scaling	0.76	0.66
**11**	Min-Max Scaling	**0.98**	**0.88**
**12**	MSC (Mean)	0.92	0.56
**13**	MSC (Median)	0.94	0.46
**14**	Multiway center	x	x
**15**	Multiway scale	0.77	0.69
**16**	Normalize	0.93	0.45

**17**	Log 10	0.95	0.62
**18**	Mean Center	x	x
**19**	Median center	0.88	0.68
**20**	Reference/Background correction	0.98	0.58

Table S3. MER quantitative values in clinical serum from ICU, patient samples, obtained with HPLC, as the reference method, and the ms-SPE to D-SERS assay.

PATIENT NO.	HPLC	*ms-SPE to D-SERS assay*
**1**	47.51 ± 0.29 µM	42.23 ± 18.71 µM
**2**	42.16 ± 4.45 µM	56.81 ± 61.15 µM
**3**	106.53 ± 40.54 µM	239.7 ± 4.45 µM
**4**	21.9 ± 0.03 µM	22.79 ± 31.56 µM
**5**	40.66 ± 0.66 µM	ND
**6**	21.11 ± 0.22 µM	ND
**7**	59.67 ± 6.22 µM	119.8 ± 73.72 µM
**8**	100.84 ± 5.23 µM	184.67 ± 51.373 µM
**9**	ND	ND
**10**	43.44 ± 0.86 µM	60.02 ± 77.63 µM
**11**	49.48 ± 1.67 µM	133.62 ± 6.0 µM
**12**	ND	ND
**13**	52.64 ± 3.22 µM	49.65 ± 78.79 µM
**14**	ND	48.24 µM
**15**	43.41 ± 1.66 µM	38.55 ± 12.11 µM
**16**	70.26 ± 0.27 µM	110.5 ± 81.18 µM
**17**	92.78 ± 1.71 µM	20.14 ± 24.83 µM
**18**	30.24 ± 1.05 µM	30.15 µM
**19**	37.34 ± 0.53 µM	ND
**20**	81.69 ± 1.36 µM	23.7 µM
**21**	118.25 ± 3.08 µM	134.83 ± 38.89 µM
**22**	304.32 ± 3.36 µM	261.56 ± 47.21 µM
**23**	75.47 ± 0.73 µM	54.6 µM
**24**	49.29 ± 2.34 µM	64.58 ± 47.55 µM
**25**	ND	ND
**26**	355.5 ± 1.5 µM	402.05 ± 120.29 µM
**27**	126.5 ± 6.0 µM	153.25 ± 80.90 µM

Table S4. Comparison of different reported methods for MER quantification

Technique	*LoD (μM)*	*Analysis Speed*	*Cost*	*Potential for On-Site/Real-Time Analysis*	*Ref.*
Microfluidics SERS	12.12	Rapid (minutes)	Moderate initial cost, low running cost	High	This work
HPLC	5.8	Moderate to Slow (hours)	High	Low	[1]
LC-MS	0.13	Moderate to Slow (hours)	Very High	Low	[2]
CE/MEKC	5.2	Moderate (hours)	Moderate	Low	[3]
Voltammetry	2.27	Rapid (minutes)	Moderate	Moderate	[4]

References:

(1) Pinder, N.; Brenner, T.; Swoboda, S.; Weigand, M. A.; Hoppe-Tichy, T. Therapeutic Drug Monitoring of Beta-Lactam Antibiotics – Influence of Sample Stability on the Analysis of Piperacillin, Meropenem, Ceftazidime and Flucloxacillin by HPLC-UV. J Pharm Biomed Anal 2017, 143, 86–93. https://doi.org/10.1016/J.JPBA.2017.05.037.

(2) Ohmori, T.; Suzuki, A.; Niwa, T.; Ushikoshi, H.; Shirai, K.; Yoshida, S.; Ogura, S.; Itoh, Y. Simultaneous Determination of Eight β-Lactam Antibiotics in Human Serum by Liquid Chromatography–Tandem Mass Spectrometry. Journal of Chromatography B 2011, 879 (15–16), 1038–1042. https://doi.org/10.1016/J.JCHROMB.2011.03.001.

(3) Chou, Y. W.; Yang, Y. H.; Chen, J. H.; Kuo, C. C.; Chen, S. H. Quantification of Meropenem in Plasma and Cerebrospinal Fluid by Micellar Electrokinetic Capillary Chromatography and Application in Bacterial Meningitis Patients. Journal of Chromatography B 2007, 856 (1–2), 294–301. https://doi.org/10.1016/J.JCHROMB.2007.06.015.

(4) Kumar Jhankal, K.; Sharma, D. K. Electrochemical Studies of Meropenem at Glassy Carbon Electrode and Its Direct Determination in Human Plasma by Square Wave Anodic Adsorptive Stripping Voltammetry. 2016, 5 (4), 1008–1018. https://doi.org/10.7598/cst2016.1292.
